# Draft Genome Sequence of *Mesorhizobium* sp. Strain SARCC-RB16n, an Effective Nodulating and Nitrogen-Fixing Symbiont of Rooibos [Aspalathus linearis (Burm. f.)] in South Africa

**DOI:** 10.1128/MRA.01187-19

**Published:** 2020-01-23

**Authors:** Ahmed Idris Hassen, Z. H. Swanevelder, Francina L. Bopape, Sandra C. Lamprecht

**Affiliations:** a Agricultural Research Council, Plant Health and Protection, Queenswood, Pretoria, South Africa; b Agricultural Research Council, Biotechnology Platform, Onderstepoort, South Africa; c Agricultural Research Council, Plant Health and Protection, Stellenbosch, South Africa; Indiana University, Bloomington

## Abstract

The draft genome sequence of *Mesorhizobium* sp. strain SARCC-RB16n reveals the presence of major symbiotic (*nod* and *nif*) and additional plant growth-promoting (PGPR) genes associated with enhanced growth of Aspalathus linearis (Burm. f.) in South Africa. The genome sequence provides vital information for the development of a commercial inoculant for rooibos cultivation.

## ANNOUNCEMENT

Aspalathus linearis (Burm. f.), the indigenous South African legume commonly referred to as rooibos, has been reported to be nodulated by members of both the alpha and beta subgroups of *Proteobacteria* (1). Among these, *Mesorhizobium* spp. have been found to be the predominant symbionts in the nodules of this legume, with successful nodulation and nitrogen fixation abilities (1, 2).

*Mesorhizobium* sp. strain SARCC-RB16n was initially isolated from the root nodule of wild rooibos growing on Cederberg Mountain, Western Cape, South Africa. The bacterial strain was first grown on yeast mannitol (YM) agar, and the purified culture was stored in 20% glycerol using multiple vials to minimize repeated freeze and thaw cycles. Glasshouse nodulation screening, together with several other strains, confirmed strain SARCC-RB16n to be the most competitive nodulator and the most effective nitrogen-fixing strain in rooibos (1).

The symbiotic properties of *Mesorhizobium* sp. strain SARCC-RB16n were further investigated by sequencing its genome to determine the underlying genetics of the nodulation and plant growth-promoting (PGPR) traits observed in the greenhouse and field trials. For DNA extraction, a single pure colony was transferred from solid YM agar into sterile yeast mannitol (YM) broth and grown for 48 hours on a rotary shaker at 28°C. DNA was extracted from 1 ml of this culture using the Wizard genomic DNA purification kit according to the manufacturer’s protocol (Promega, Madison, WI, USA). Paired-end DNA libraries were prepared using the Nextera protocol (Illumina, USA) and were then sequenced on an Illumina HiSeq 2500 instrument at the Agricultural Research Council’s Biotechnology Platform (Onderstepoort, South Africa). A total of more than 32 million paired sequences (125 bp each) were obtained prior to adaptor and quality trimming and the merging of overlapped reads. CLC Genomics Workbench 8.5.1 was used for the trimming and genome assembling, with the low-quality sequence limit set to 0.05, removing >2 ambiguous nucleotides, sequencing adaptors, and Nextera transposases in a sequence and disregarding sequences of 50 bp or less. This resulted in a final number of more than 31 million paired-end trimmed and quality-checked reads, which were used to merge overlapping reads (just over 17.2 million overlapped reads). Both merged and unmerged reads, with a word size of 35, were used for the final *de novo* genome assembly. This resulted in a genome size of 7,145,923 bp with a GC content of 66.2%, 84 contigs, an *N*_50_ value of 217,074 bp (including scaffolds), and an *L*_50_ value of 17. Genome annotation was performed using the NCBI Prokaryotic Genome Annotation Pipeline (PGAP) (3, 4) and resulted in a total of 7,412 coding sequences and 52 RNA copies (3 rRNAs and 49 tRNAs).

The functional nitrogen fixation (*nif*) genes found on contig 24 are all part of the subsystem and include *nifA*, *nifB*, *nifH*, *nifX*, *nifE*, *nifF*, *nifW*, *nifQ*, *nifZ*, *nifT*, *nifN*, and *nifS*. On this same contig are found the nodulation (*nod*) genes, which comprise *nodA*, *nodC*, *nodD*, and *nodJ*. The annotation also detected beneficial PGPR genes that include the *acdS* gene that codes for 1-aminocyclopropane-1-carboxylate (ACC) deaminase, genes for auxin biosynthesis and phosphate solubilization, and genes that code for the production of phenazine antibiotics.

### Data availability.

This whole-genome shotgun project has been deposited at DDBJ/ENA/GenBank under the accession number PXXM00000000. The version described in this paper is PXXM01000000. The SRA accession number is PRJNA438233.
